# Coronary Artery Disease Is Associated with an Increased Amount of T Lymphocytes in Human Epicardial Adipose Tissue

**DOI:** 10.1155/2019/4075086

**Published:** 2019-02-07

**Authors:** Miloš Mráz, Anna Cinkajzlová, Jana Kloučková, Zdeňka Lacinová, Helena Kratochvílová, Michal Lipš, Michal Pořízka, Petr Kopecký, Aneta Pierzynová, Tomáš Kučera, Vojtěch Melenovský, Ilja Stříž, Jaroslav Lindner, Martin Haluzík

**Affiliations:** ^1^Diabetes Centre, Institute for Clinical and Experimental Medicine, Prague, Czech Republic; ^2^Department of Medical Biochemistry and Laboratory Diagnostics, First Faculty of Medicine, Charles University and General University Hospital, Prague, Czech Republic; ^3^Centre for Experimental Medicine, Institute for Clinical and Experimental Medicine, Prague, Czech Republic; ^4^Department of Anaesthesiology, Resuscitation and Intensive Medicine, First Faculty of Medicine, Charles University and General University Hospital, Prague, Czech Republic; ^5^Institute of Histology and Embryology, First Faculty of Medicine, Charles University, Prague, Czech Republic; ^6^Cardiology Department, Cardiac Centre, Institute for Clinical and Experimental Medicine, Prague, Czech Republic; ^7^Department of Clinical and Transplantation Immunology, Institute for Clinical and Experimental Medicine, Prague, Czech Republic; ^8^2nd Department of Surgery-Department of Cardiovascular Surgery, Charles University and General University Hospital, Prague, Czech Republic

## Abstract

Immunocompetent cells including lymphocytes play a key role in the development of adipose tissue inflammation and obesity-related cardiovascular complications. The aim of the study was to explore the relationship between epicardial adipose tissue lymphocytes and coronary artery disease (CAD). To this end, we studied the content and phenotype of lymphocytes in peripheral blood, subcutaneous adipose tissue (SAT), and epicardial adipose tissue (EAT) in subjects with and without CAD undergoing elective cardiac surgery. Eleven subjects without CAD (non-CAD group) and 22 age-, BMI-, and HbA_1C_-matched individuals with CAD were included into the study. Blood, SAT, and EAT samples were obtained at the beginning of surgery. Lymphocyte populations were quantified as % of CD45+ cells using flow cytometry. Subjects with CAD had a higher total lymphocyte amount in EAT compared with SAT (32.24 ± 7.45 vs. 11.22 ± 1.34%, *p* = 0.025) with a similar trend observed in non-CAD subjects (29.68 ± 7.61 vs. 10.13 ± 2.01%, *p* = 0.067). T (CD3+) cells were increased (75.33 ± 2.18 vs. 65.24 ± 4.49%, *p* = 0.032) and CD3- cells decreased (21.17 ± 2.26 vs. 31.64 ± 4.40%, *p* = 0.028) in EAT of CAD relative to the non-CAD group. In both groups, EAT showed an elevated percentage of B cells (5.22 ± 2.43 vs. 0.96 ± 0.21%, *p* = 0.039 for CAD and 12.49 ± 5.83 vs. 1.16 ± 0.19%, *p* = 0.016 for non-CAD) and reduced natural killer (NK) cells (5.96 ± 1.32 vs. 13.22 ± 2.10%, *p* = 0.012 for CAD and 5.32 ± 1.97 vs. 13.81 ± 2.72%, *p* = 0.022 for non-CAD) relative to SAT. In conclusion, epicardial adipose tissue in subjects with CAD shows an increased amount of T lymphocytes relative to non-CAD individuals as well as a higher number of total and B lymphocytes and reduced NK cells as compared with corresponding SAT. These changes could contribute to the development of local inflammation and coronary atherosclerosis.

## 1. Introduction

Excessive accumulation of adipose tissue has long been associated with chronic low-grade inflammation leading to accelerated atherosclerosis and the development of cardiovascular diseases (CVD) [1, 2]. Although increased systemic production of proinflammatory adipocytokines from the metabolically highly active visceral adipose tissue depot is considered the main effector of obesity-related inflammation, recent data indicate the importance of local perivascular adipose tissue depots and their paracrine-acting products in the process of vascular atherogenesis with a pivotal role played by the epicardial adipose tissue (EAT) due to its proximity to coronary arteries [3–5]. EAT is localized predominantly in the atrioventricular and interventricular grooves and free wall of the right ventricle thus being in intimate contact with the coronaries. The paracrine effect of its products is further underscored by the absence of fascia between EAT and underlying tissues [6, 7]. Moreover, EAT mass was shown to correlate with the risk of type 2 diabetes mellitus (T2DM) and CVD [8, 9]. Several studies also reported increased mRNA expression of proinflammatory adipocytokines in EAT of subjects with coronary artery disease (CAD) compared with subcutaneous fat as well as with EAT of non-CAD individuals [10–14].

The main mechanism responsible for the development of adipose tissue inflammation is its infiltration by circulating immune cells [15, 16]. M1-polarized proinflammatory macrophages were suggested to play a leading role in these processes; however, the initiation of macrophage recruitment into adipose tissue and their particular polarization state (proinflammatory M1 versus anti-inflammatory M2) seem to be triggered by other immune cells, especially lymphocytes [17–19]. Different lymphocyte subpopulations were shown to be present in human adipose tissue with T helper (Th) lymphocytes capable of producing pro- as well as anti-inflammatory factors being the most abundant [18, 20]. T cytotoxic cells associated with M1 polarization were represented more in visceral compared with subcutaneous adipose tissue (SAT) [21]. Other lymphoid precursor-derived immune cells found in adipose tissue include natural killer (NK) and natural killer T (NKT) cells and B lymphocytes [22].

Despite the available data on adipose tissue lymphocytes, little is known about their presence in EAT. The occurrence of T lymphocytes (CD3+ cells) in EAT of CAD subjects was directly confirmed by immunohistochemistry, while the detection of IgG and IgM antibodies in epicardial fat samples also indicates the presence of B lymphocytes [11, 13, 23]. However, a more comprehensive assessment of different lymphocyte subpopulations in EAT as well as their relation with CAD is still lacking. To this end, we performed a complex cytometric and histological analyses of lymphocyte subtypes in peripheral blood and subcutaneous and epicardial adipose tissues of subjects with and without coronary artery disease undergoing elective cardiac surgery.

## 2. Patients and Methods

### 2.1. Study Subjects

Eleven subjects without CAD and 22 individuals with CAD, all undergoing elective cardiac surgery, were included into the study. Written informed consent was signed by each subject prior to inclusion, and the study was approved by the Human Ethics Review Board, First Faculty of Medicine and General University Hospital, Prague, Czech Republic. The study was performed in accordance with the principles of the Declaration of Helsinki as revised in 2008.

Adult age and coronary artery disease or valvular disorder scheduled for elective coronary artery bypass graft implantation, valvular replacement, or valvuloplasty were selected as study inclusion criteria, while all acute cardiosurgical procedures along with the inability or refusal to provide written informed consent were defined as exclusion criteria.

### 2.2. Blood and Adipose Tissue Sampling

Blood samples were taken before the beginning of surgery after overnight fasting. Samples were centrifuged for 10 min at 1000 × g within 30 min after withdrawal. Serum or plasma aliquots were subsequently stored at -80°C.

Analogously, 1-2 g of SAT and EAT was obtained at the beginning of surgery immediately after sternotomy. EAT was taken from the anterior interventricular sulcus or the right margin of the heart, and SAT was obtained from the sternotomy site. Freshly collected specimens in PBS buffer (0.01 M PBS, pH 7.4) were used for flow cytometry, and aliquots in RNAlater® solution (Ambion®-Invitrogen, Carlsbad, California, USA) were stored at -80°C and subsequently used for determination of mRNA expression. Samples for immunohistochemistry were immediately fixed in 4% formaldehyde and processed further.

### 2.3. Hormonal and Biochemical Assays

Serum levels of cytokines were measured by the multiplex assay MILLIPLEX® MAP Human Cytokine/Chemokine Magnetic Bead Panel (Merck KGaA, Darmstadt, Germany). Sensitivity for IFN-*γ* was 0.8 pg/ml, for IL-10 1.1 pg/ml, for IL-6 0.9 pg/ml, for IL-8 0.4 pg/ml, and for TNF-*α* 0.7 pg/ml. The intra- and interassay variabilities for all analytes were between 5.0 and 15.0%. Serum high sensitive C-reactive protein (hsCRP) levels were measured by the high sensitive ELISA kit (Bender MedSystems, Vienna, Austria) with a sensitivity of 3 pg/ml. Sensitivity was 7 ng/ml. The intra- and interassay variabilities for all assays were between 5.0 and 10.0%.

Biochemical parameters were measured, and LDL cholesterol was calculated at the Department of Medical Biochemistry and Laboratory Diagnostics, General University Hospital, Prague, Czech Republic, by standard laboratory methods.

### 2.4. Quantitative Real-Time PCR

Samples of adipose tissue were homogenized on MagNA Lyser Instrument (Roche Diagnostics GmbH, Mannheim, Germany). The total RNA was extracted on the MagNA Pure instrument using the Magna Pure Compact RNA Isolation kit (tissue) (Roche Diagnostics GmbH, Mannheim, Germany). RNA concentration was determined from absorbance at 260 nm on NanoPhotometer (Implen, Munchen, Germany). Reverse transcription was performed using random primers according to the manufacturer's protocol of the High-Capacity cDNA Reverse Transcription Kits (Applied Biosystems, Foster City, CA, USA). The input amount of RNA was 250 *μ*g per reaction. The determination of gene expression was performed on the 7500 Real-Time PCR System (Applied Biosystems, Foster City, CA, USA). For reaction, a mix of TaqMan® Universal PCR Master Mix II, NO AmpErase® UNG (Applied Biosystems, Foster City, CA, USA), nuclease-free water (Fermentas Life Science, Vilnius, Lithuania), and specific TaqMan® Gene Expression Assays (Applied Biosystems, Foster City, CA, USA) was used. Beta-2-microglobulin was used as endogenous reference. Relative gene expression was calculated using the formula 2^-ΔΔCt^.

### 2.5. Isolation of Stromal Vascular Fraction from Adipose Tissue and Flow Cytometry

The standard 0.5-1.0 g amount of adipose tissue was minced with sterile scissors, and visible blood vessels were removed. Samples were washed in PBS, digested by 0.01% collagenase (Collagenase from Clostridium histolyticum, St. Louis, MO, USA) for 30 min at 37°C and centrifuged for 12 min at 1200 × g. Visible adipocytes were then manually collected from the surface via a pipette with subsequent repeated washings and removal of remaining adipocytes from the supernatant. Finally, samples were filtered through Falcon® 40 *μ*m Cell Strainer (Becton, Dickinson and Company, Franklin Lakes, USA) to eliminate any remnant adipocytes.

**Figure 1:**
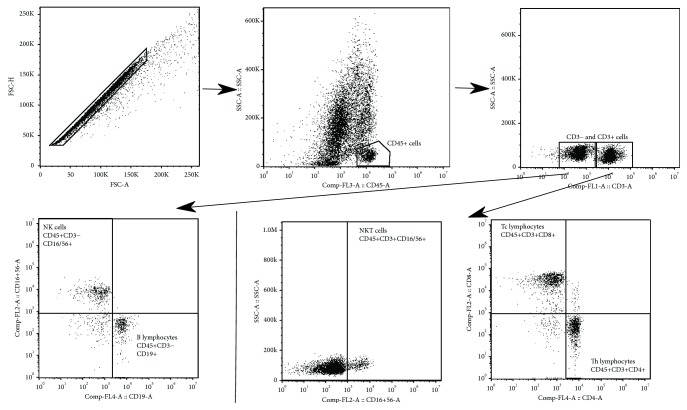
Flow cytometry: gating strategy. Doublets were excluded, lymphocytes were gated according to SSC properties and CD45 positivity, and T (CD3+) and CD3- cells were assessed. B lymphocytes (CD19+CD3-CD45+ cells) and NK cells (CD16/56+CD3-CD45+ cells) were gated from CD3- cells. T helper lymphocytes (CD4+CD3+CD45+ cells), T cytotoxic lymphocytes (CD8+CD3+CD45+ cells), and NKT cells (CD16/56+CD3+CD45+ cells) were gated from T (CD3+) cells.

Flow cytometry was performed using freshly isolated and filtered stromal vascular fraction or EDTA whole blood. A total amount of 100 *μ*l of cell suspension with average 10^6^ cell content were labeled by monoclonal antibodies conjugated with FITC (fluorescein isothiocyanate), PE (phycoerythrin), PerCP (peridinin-chlorophyll protein complex), and APC (allophycocyanin). For labeling a commercial KOMBITEST™ CD3 FITC, CD8 PE, CD45 PerCP, CD4 APC and KOMBITEST™ CD3 FITC, CD16+56 PE, CD45 PerCP, CD19 APC (Exbio Prague, a.s., Vestec, Czech Republic) was used. The samples were labeled in the dark for 30 min at 2–8°C, and then red cells were lysed using Excellyse I (Exbio Prague, a.s., Vestec, Czech Republic) according to manufacturer's instructions. Finally, labeled cells were analyzed on BD Accuri™ C6 (Becton, Dickinson and Company, Franklin Lakes, USA). Data analysis was performed using FlowJo X 10.0.7r2 software (FlowJo, LCC, Ashland, USA). The gating strategy was as follows: doublets were excluded, lymphocytes were gated according to SSC properties and CD45 positivity, and then CD3- and CD3+ cells were assessed (Figure 1). B lymphocytes (CD19+CD3-CD45+ cells) and NK cells (CD16/56+CD3-CD45+ cells) were gated from CD3- cells. T helper lymphocytes (CD4+CD3+CD45+ cells), T cytotoxic lymphocytes (CD8+CD3+CD45+ cells), and NKT cells (CD16/56+CD3+CD45+ cells) were gated from CD3+ cells (Figure 1). CD45+ lymphocytes were expressed as the percentage of single cells and other lymphocyte populations as the percentage of CD45+ lymphocytes. The percentage of gated CD45+ lymphocytes, T lymphocytes, and CD3- cells from both KOMBITEST™ were statistically compared (data not shown), and only data from one KOMBITEST™ are presented. The minimal count of acquired events was 50,000.

### 2.6. Immunohistochemistry

Samples of SAT and EAT were immediately fixed in 4% formaldehyde, processed, embedded in paraffin, sectioned, and stained with hematoxylin-eosin. The type, amount, and integrity of tissue were determined, and in accordance with this evaluation, the specimens were used for immunohistochemistry.

For an indirect immunohistochemical method, 7 *μ*m thick sections were used. The sections were deparaffinized and immersed into preheated antigen-retrieval solution with pH 8.5 (Tris-chelaton III), incubated at 98°C for 10 min, and allowed to cool to room temperature. The rabbit polyclonal anti-human CD3 antibody (ab5690, Abcam, United Kingdom) was used as the primary antibody (dilution 1 : 4000). Visualization was achieved using LSAB+ Dako REAL™ Detection System, Peroxidase/DAB+, Rabbit/Mouse according to previous publications [24]. The nuclei were counterstained by Harris's hematoxylin. Control samples of the human tonsil underwent the same staining (data not shown). Omission of the primary antibody gave the negative result.

For quantification, 20 images were collected via systematic random sampling from each tissue section using the 40x dry objective of the Leica DMLB microscope (Leica Microsystems GmbH, Wetzlar, Germany). The number of lymphocytes was assessed by manual analysis of microscopic images using ImageJ 1.50i software (National Institutes of Health, USA). Dense connective tissue, empty fields, and bigger blood vessels were excluded from the area of a given image.

### 2.7. Statistical Analysis

Statistical analysis was performed, and graphs were created using SigmaPlot 13.0 (SPSS Inc., Chicago, IL, USA). Results are expressed as mean ± standard error of the mean (SEM). One-way ANOVA followed by the Holm-Sidak test, one-way ANOVA on ranks followed by Dunn's method, unpaired *t*-test or the Mann–Whitney rank sum test, and the paired test or Wilcoxon signed-rank test were used for the assessment of intergroup differences, as appropriate. The Spearman or Pearson correlation test was used to assess the association between lymphocytes and other measured parameters. Baseline data of all study subjects were used for correlation analyses. Statistical significance was assigned to *p* < 0.05.

## 3. Results

### 3.1. Anthropometric and Biochemical Parameters

No differences in baseline demographic, anthropometrical, and biochemical parameters including body mass index (BMI), fasting glucose and HbA_1C_, lipid profile, and renal function were observed between CAD and non-CAD groups (Table 1). Arterial hypertension was more common in CAD subjects, whereas no difference in the occurrence of diabetes mellitus was seen between both groups (Table 1). As expected, in the CAD group, the prevalent surgery type was coronary artery bypass grafting (CABG), while non-CAD subjects underwent exclusive valvular surgery.

### 3.2. Circulating Cytokines

At baseline, subjects with CAD showed elevated circulating levels of TNF-*α* and IL-6 and reduced IL-23 relative to the non-CAD group, while no difference was present in other cytokines (Table 2).

### 3.3. mRNA Expression in EAT and SAT

No difference in mRNA expression of the evaluated cytokines, lymphocyte-attracting chemokines, and their receptors was found between CAD and non-CAD subjects as well as between different adipose tissue pools (SAT vs. EAT), although a tendency to higher levels of IL-6 was present in EAT of both groups (Table 3).

### 3.4. Lymphocyte Subpopulations in Peripheral Blood, SAT, and EAT

#### 3.4.1. Flow Cytometry

**Figure 2:**
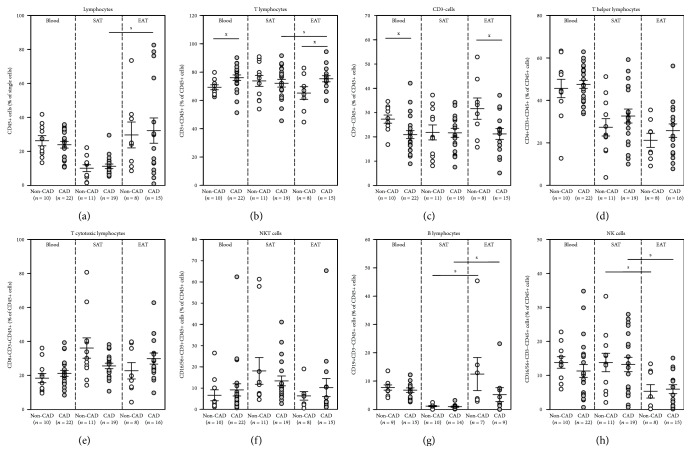
Different lymphocyte populations in peripheral blood and subcutaneous and epicardial adipose tissues of subjects with and without coronary artery disease—flow cytometry. (a) Total lymphocytes (CD45+ cells), (b) T lymphocytes (CD3+ CD45+ cells), (c) CD3- cells (CD3-CD45+ cells), (d) T helper lymphocytes (CD4+CD3+CD45+ cells), (e) T cytotoxic lymphocytes (CD8+CD3+CD45+ cells), (f) NKT cells (CD16/56+CD3+CD45+ cells), (g) B lymphocytes (CD19+CD3-CD45+ cells), and (h) NK cells (CD16/56+CD3-CD45+ cells). CD45+ cells are represented as the percentage of single cells; all other cell subtypes are represented as the percentage of CD45+ cells. Circles represent individual subjects while bars show mean ± SEM. ^X^*p* < 0.05 vs. without CAD, ^S^*p* < 0.05 vs. subcutaneous adipose tissue. SAT: subcutaneous adipose tissue; EAT: epicardial adipose tissue; non-CAD subjects: white circles; CAD subjects: grey circles.

In peripheral blood, neither the total number of circulating lymphocytes nor their different subpopulations (T helper, T cytotoxic, NKT and NK cells, and B lymphocytes) showed any difference between both study groups (Figure 2). Subjects with CAD had a higher lymphocyte amount in EAT compared with SAT (32.24 ± 7.45 vs. 11.22 ± 1.34%, *p* = 0.025) with a similar trend observed in non-CAD subjects (29.68 ± 7.61 vs. 10.13 ± 2.01%, *p* = 0.067)—Figure 2(a). In EAT of CAD subjects, T cells (CD3+) were increased and CD3- cells were decreased relative to EAT of non-CAD individuals (CD3+: 75.33 ± 2.18 for CAD vs. 65.24 ± 4.49% for non-CAD, *p* = 0.032; CD3-: 21.17 ± 2.26 for CAD vs. 31.64 ± 4.40% for non-CAD, *p* = 0.028) as well as compared with corresponding SAT (75.33 ± 2.18 for EAT vs. 72.18 ± 2.71% for SAT, *p* = 0.033) (Figures 2(b) and 2(c)). In both groups, EAT showed reduced percentage of NK cells and elevated B cells relative to SAT (NK cells: 5.96 ± 1.32 vs. 13.22 ± 2.10%, *p* = 0.012 for CAD and 5.32 ± 1.97 vs. 13.81 ± 2.72%, *p* = 0.022 for non-CAD and B cells: 5.22 ± 2.43 vs. 0.96 ± 0.21%, *p* = 0.039 for CAD and 12.49 ± 5.83 vs. 1.16 ± 0.19%, *p* = 0.016 for non-CAD). No significant difference between CAD and non-CAD subjects was seen in any of the T, B, or NK cell subpopulations in either SAT or EAT (Figures 2(d)–2(h)).

#### 3.4.2. Immunohistochemistry

**Figure 3:**
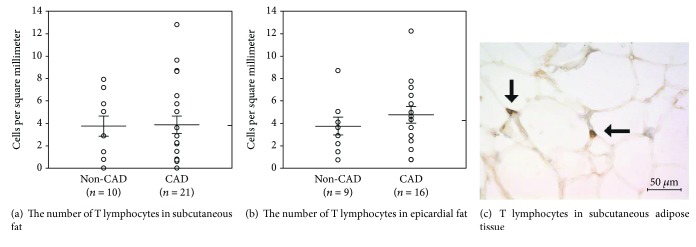
T lymphocytes (CD3+ cells) in subcutaneous and epicardial adipose tissues of subjects with and without coronary artery disease—immunohistochemistry. (a) Subcutaneous adipose tissue, (b) epicardial adipose tissue, (c) T lymphocytes in subcutaneous adipose tissue of a nonobese patient without T2DM and CAD (black arrows, magnification 40x). Data are shown as the number of T cells per mm^2^. Circles represent individual subjects while bars show mean ± SEM. CAD: coronary artery disease; T2DM: type 2 diabetes mellitus.

T (CD3+) lymphocytes were mostly found as single scattered cells in the interstitial tissue surrounding adipocytes and tended to be more abundant in EAT compared to SAT. The following tendencies were detected: T (CD3+) lymphocytes were more abundant in EAT of CAD relative to non-CAD subjects as well as to corresponding SAT (Figure 3). Interestingly, the number of T cells in EAT tended to be slightly higher with increased BMI, while the presence of T2DM had opposite effect. By contrast, in SAT, increased frequency of T lymphocytes was associated with both obesity and T2DM (data not shown).

## 4. Discussion

The most important finding of this study is the higher T cell content in epicardial adipose tissue of subjects with coronary artery disease as compared with individuals without CAD. We further found increased amount of total lymphocytes and B cells and reduced NK cells in epicardial relative to subcutaneous adipose tissue regardless of CAD.

Adipose tissue immune cells are considered one of the main drivers of local as well as systemic low-grade inflammation, which links obesity with type 2 diabetes mellitus and accelerated atherosclerosis [15, 22]. The proinflammatory and proatherogenic effects of adipose tissue immune cells are caused not only by their products released into circulation but also by their direct paracrine action, especially in adipose tissue pools lying in the vicinity of large blood vessels, i.e., perivascular and epicardial adipose tissues [3, 5, 25]. Epicardial adipose tissue was shown to be more proinflammatory compared with subcutaneous fat due to increased production of proinflammatory adipokines and higher degree of oxidative stress and was suggested to play a role in the development of coronary atherosclerosis, as its volume correlates with the rate of coronary events regardless of traditional cardiovascular risk factors [8, 10–13]. To date, only limited data on the amount and composition of immune cells in EAT are available in humans focusing primarily on macrophages as the main proinflammatory effector cells [26]. However, lymphocytes constitute the second largest immune cell population in adipose tissue and were suggested as one of the initiators of macrophage and other immune cell recruitment into adipose tissue [18]. The presence of lymphocytes in EAT has already been confirmed previously by immunochemistry [13]. Here, we show that EAT contains a significantly larger amount of lymphocytes than SAT, and this difference is further augmented by the presence of coronary artery disease. These findings are in line with the reported higher expression of the lymphocyte marker CD45 in EAT compared with SAT [11, 23].

T lymphocytes represent the main lymphocyte subpopulation in adipose tissue [18]. Similar to macrophages, T lymphocytes are able to differentiate into pro- and anti-inflammatory phenotypes. In animals, the proinflammatory Th1 cells were shown to induce M1 polarization of adipose tissue macrophages, mainly through the production of INF-*γ* [19]. In contrast, the IL-4- and TGF-*β*-producing Th2 cells switch the macrophage phenotype to the anti-inflammatory M2 type [27]. T cytotoxic (CD8+) cells are also associated with M1 macrophage polarization and were shown to be increased in visceral relative to subcutaneous adipose tissue, while natural killer T (NKT) cells are capable of producing both pro- and anti-inflammatory factors [21, 28]. In this study, we found increased percentage of T cells in EAT of subjects with CAD as compared to without CAD using flow cytometry, and this finding was further confirmed by immunohistochemistry. However, we did not find any significant difference in any of the examined T cell subpopulations either between CAD and non-CAD subjects or between EAT and SAT, which was most probably caused by the relatively low number of obtained samples and heterogeneity of epicardial adipose tissue.

The occurrence of B lymphocytes in EAT has previously been suggested by positive immunoglobulin detection [13]. Here, we verify the presence of B cells in human EAT, where they take up a markedly higher percentage of total lymphocytes than in SAT irrespective of CAD. These findings are in line with the recent data by Horckmans et al. who identified larger clusters of B and T lymphocytes in EAT of subjects with CAD as compared to CAD-free controls [29]. Aside from immunoglobulin production, B lymphocytes may also contribute to the initiation and propagation of inflammatory reactions due to their antigen-presenting properties [30]. Interestingly, the last lymphocyte subtype analyzed in our study—NK cells—was reduced in EAT of both CAD and non-CAD groups, which seems in contrast to their previously demonstrated association with macrophage infiltration and M1 polarization and requires further investigation [31].

Surprisingly, the presence of type 2 diabetes mellitus had no significant effect on any of the studied lymphocyte subpopulations in EAT or SAT of either CAD or non-CAD subjects. This might be most probably caused by the small number of subjects in this subanalysis or their excellent glucose control. Nevertheless, in EAT, T cytotoxic and NKT cells correlated positively with fasting glucose (*R* = 0.531, *p* = 0.008 for T cytotoxic and *R* = 0.414, *p* = 0.049 for NKT cells) and HbA_1C_ (*R* = 0.420, *p* = 0.046 for T cytotoxic and *R* = 0.555, *p* = 0.008 for NKT cells) indicating a more proinflammatory EAT phenotype in subjects with inadequately controlled T2DM. Obesity was associated with increased numbers of CD3+, CD8+, and NK cells in EAT of non-CAD subjects, while these differences were not present in individuals with CAD (data not shown) suggesting a more deleterious effect of obesity on EAT in the absence of coronary atherosclerosis.

Systemic and local low-grade inflammations are established risk factors for the development of atherosclerosis and coronary artery disease [32]. This was confirmed in our study not only by increased levels of hsCRP but also by elevated circulating concentrations of proinflammatory cytokines TNF-*α* and IL-6 in subjects with CAD. In contrast, systemic levels of IL-23 associated with autoimmune processes were reduced in CAD relative to the non-CAD group. One might speculate that reduced IL-23 could facilitate the transition of Th phenotype from Th17 to Th1 in CAD subjects, as IL-23 is involved in the induction of Th17 lymphocytes [33]. Somehow unexpectedly, mRNA expression of TNF-*α* and IL-6 along with the chemokine CCL-5 responsible for the chemotaxis of lymphocytes and its receptor CCR-1 did not differ between CAD and non-CAD subjects or between SAT and EAT, respectively, even though a trend to increased mRNA expression of IL-6 was observed in EAT vs. SAT in both groups.

The main limitations of our study include the relatively low sample size, especially in the non-CAD group, and preexisting differences in several baseline characteristics between both groups including the prevalence of arterial hypertension and CABG as compared with valvular surgery, which are nevertheless the consequences of the principal pathological condition studied (coronary artery disease). Moreover, the presence of a small amount of coronary atherosclerosis in non-CAD subjects cannot be completely ruled out. This plus the inclusion of patients with valve surgery in the CAD group could potentially diminish the differences between the CAD and non-CAD groups. Two different EAT sampling sites—anterior interventricular sulcus and right cardiac margin—could have also influenced the results as the periventricular and pericoronary location of EAT was recently shown to have different transcriptomic signatures [34]; however, sampling location in our study was guided primarily by the availability of EAT and thus could not be kept absolutely uniform in all participants. Finally, the cross-sectional character of our study does not enable us to determine whether the observed changes in EAT lymphocyte content directly participate in the development of CAD or if they are solely a marker of coronary atherosclerosis. Prospective studies using interventions able to modify the number and/or function of different lymphocyte subpopulations are required to clarify this issue.

## 5. Conclusions

Taken together, our study demonstrated increased T lymphocyte content in epicardial adipose tissue of subjects with coronary artery disease along with an elevated percentage of the total and B lymphocytes and a reduced number of NK cells in EAT of both CAD and non-CAD individuals. These data suggest a potential role for epicardial adipose tissue lymphocytes in the development and/or progression of coronary artery disease.

## Figures and Tables

**Table 1:** Baseline characteristics of study subjects.

	Non-CAD	CAD
Number of subjects (females/males)	11 (4/7)	22 (5/17)
Age (year)	63.3 ± 3.8	67.9 ± 1.8
BMI (kg/m^2^)	30.9 ± 1.5	28.9 ± 1.0
Creatinine (*μ*mol/l)	68.63 ± 4.55	78.50 ± 3.94
Fasting glycemia (mmol/l)	6.58 ± 0.88	6.97 ± 0.37
HbA_1c_ (mmol/mol)	41.73 ± 3.85	43.29 ± 2.27
Total cholesterol (mmol/l)	4.36 ± 0.34	3.98 ± 0.19
Triglycerides (mmol/l)	1.69 ± 0.21	1.48 ± 0.22
hs C-reactive protein (mg/ml)	3.85 ± 0.84	9.44 ± 2.51
Lymphocyte count		
Absolute (10^9/l)	1.43 ± 0.15	1.43 ± 0.10
Relative (%)	26.66 ± 2.57	22.65 ± 1.71
Diabetes mellitus (*n*, %)	4 (36.4)	11 (50)
Arterial hypertension (*n*, %)	7 (63.6)	21 (95.5)^a^
LV EF (%)	58.8 ± 3.3	50.7 ± 3.7
Operation type		
CABG (*n*, %)	0 (0)	16 (72.7)^a^
Valve replacement or valvuloplasty (*n*, %)	11 (100)	13 (59.1)^a^

Data are mean ± SEM. ^a^*p* < 0.05 vs. without CAD. LV EF: left ventricular ejection fraction; CABG: coronary artery bypass graft.

**Table 2:** The influence of coronary artery disease on circulating cytokine levels.

	Non-CAD (*n* = 11)	CAD (*n* = 22)
TNF-*α* (pg/ml)	7.41 ± 1.40	12.25 ± 1.50^a^
IFN-*γ* (pg/ml)	28.92 ± 25.25	35.53 ± 18.72
IL-6 (pg/ml)	0.60 ± 0.03	7.17 ± 2.23^a^
IL-8 (pg/ml)	10.03 ± 4.31	12.02 ± 4.04
IL-10 (pg/ml)	11.10 ± 3.39	14.31 ± 5.56
IL-23 (pg/ml)	496.06 ± 82.26	325.61 ± 92.72^a^

Data are mean ± SEM. ^a^*p* < 0.05 vs. without CAD.

**Table 3:** The influence of coronary artery disease on mRNA expression of selected cytokines/chemokines and lymphocyte markers in subcutaneous and epicardial adipose tissues.

	Non-CAD (*n* = 11)	CAD (*n* = 22)
	Subcutaneous adipose tissue	
Adiponectin	1.04 ± 0.22	1.06 ± 0.06
CCL-5	1.12 ± 0.23	1.16 ± 0.16
CCR-1	1.04 ± 0.12	1.27 ± 0.21
IL-6	0.97 ± 0.15	1.48 ± 0.34
TNF-*α*	1.04 ± 0.15	1.29 ± 0.17

	Epicardial adipose tissue	
Adiponectin	1.00 ± 0.12	1.04 ± 0.13
CCL-5	1.08 ± 0.10	1.10 ± 0.12
CCR-1	0.97 ± 0.10	1.21 ± 0.17
IL-6	3.40 ± 2.20	4.93 ± 3.09
TNF-*α*	1.12 ± 0.21	1.88 ± 0.50

Data are mean ± SEM. ^a^*p* < 0.05 vs. subcutaneous adipose tissue.

## Data Availability

The data used to support the findings of this study are available from the corresponding author upon request.
